# A Breast Cancer Prediction Model Based on a Panel from Circulating Exosomal miRNAs

**DOI:** 10.1155/2022/5170261

**Published:** 2022-10-20

**Authors:** Yangyang Pan, Xiaoli Xu, Ting Luo, Shuqing Yang, Dan Zhou, Yan Zeng

**Affiliations:** ^1^Precision Clinical Laboratory, Central People's Hospital of Zhanjiang, Zhanjiang, Guangdong, China; ^2^Department of Hematology, First People's Hospital of Foshan, Foshan, Guangdong, China; ^3^Key Laboratory of Xinjiang Endemic and Ethnic Diseases, School of Medicine, Shihezi University, Shihezi, Xinjiang, China; ^4^Breast Center, First People's Hospital of Foshan, Foshan, Guangdong, China

## Abstract

Breast cancer (BC) has been a serious threat to women's health. Exosomes contain a variety of biomolecules, which is an excellent choice as disease diagnostic markers, but whether it could be applied as a noninvasive biomarker for BC diagnosis demands to be additional studied. In this study, we aimed at creating a predictive model and reveal the value of plasma exosomal miRNA (exo-miRNA) in early diagnosis of BC. Firstly, exosomes isolated from plasma were identified by Nanoparticle Tracking Analysis (NTA), Transmission Electron Microscope (TEM), and Western Blot. miRNA expression in plasma samples from 56 BC patients and 40 normal controls was analyzed by high-throughput sequencing. miRNAs with strong correlation characteristics were selected by Lasso logistic regression. Then, we built the training set and test set, evaluated the Lasso regression accuracy, and evaluated the performance of different models in the training set and test set. Finally, GO analysis, KEGG, and Reactome pathway enrichment analysis were used to understand the biological significance of 16 characteristic miRNAs. The successful separation of exosomes in serum was identified by NTA, TEM, and Western Blot. The training set data matrix containing 1962 miRNAs was obtained by sequencing for model construction, and 16 strongly correlated miRNAs were selected by Lasso logistic regression. The accuracy of Lasso regression in training set and test set were 97.22% and 95.83%, respectively. We built different models and evaluated the performance of each model in the training set and test set. The results showed that the AUC values of Lasso, SVM, GBDT, and Random Forest model in the training set were 1, and the AUC values in the test set were 0.979, 0.936, 0.971, and 0.979, respectively. Bioinformatics analysis showed that 16 signature miRNAs were significantly enriched in cancer-related pathways such as herpes simplex virus 1 infection, TGF-*β* signaling, and Toll-like receptor family. The results of this study suggest that the 16 characteristic miRNAs screened from plasma exosomes can be used as a group of biomarkers, and the prediction model constructed based on this set of markers is expected to be used in the early diagnosis of BC.

## 1. Introduction

As the highly heterogeneous malignancies at the molecular level, the morbidity and mortality rate of breast cancer (BC) are very high and increasing year by year. In 2018, the incidence rate of cancer in the United States displayed that about 266 thousand of new cases of BC accounted for 30% of all women's malignant tumors, far exceeding the second lung cancer (13%) and the third colorectal cancer (7%) [1]. Experts pointed out that the cure rate of the disease can reach more than 90% if diagnosed and treated as soon as possible. At present, BC screening methods include B-mode ultrasound, mammography, magnetic resonance imaging, and biopsy. However, these methods are low sensitivity, radiative, expensive, or invasive, which makes the diagnosis of BC still lags behind [2–5]. Most patients missed the best treatment period at the time of diagnosis, which indicates that there is a great demand for effective early diagnosis methods in clinic. Exosome is an attractive source of biomarker for disease detection and prognosis because of their selective enrichment and ease of accessibility from biofluids [6]. Saliva and urine exosomal proteins have already been proposed as biomarkers for non-small lung carcinoma, prostate cancer, and gastric cancer [7–9]. Moreover, plasma exosome is a source of early disease biomarker for Alzheimer's and Parkinson's neurological diseases [10, 11].

Exosomes are a kind of membrane vesicles with a diameter of about 30~150 nm, which can come from diversified cells, are closely related to multiple biological processes [12], and are important tools for material and information exchange between cells. The size of miRNA (miRNA) is usually about 20 nucleotides. Studies have confirmed that the regulation of fertility in mammals is inseparable from miRNA, while exosomes can carry miRNA to remote cells to modulate cell physiological activities and its double-layer membrane structure can ensure the stability of miRNA in body fluids [13]. Therefore, exosomal miRNA (exo-miRNA) is suitable for the study of diagnostic markers and pathogenesis of diseases. Studies have shown that the detection of miR-1246, miR-21 [14], miR-373 [15], or miR-423-5p [16] in plasma exosome can be used to diagnose BC. However, the sensitivity and specificity of single miRNA detection in disease diagnosis is relatively low. Combined detection of multiple miRNAs may effectively improve the detection rate of BC. Therefore, developing a set of exo-miRNAs which can be used for BC diagnosis is a vital task.

In this study, after determining the success of plasma exocrine separation, we analyzed the expression of miRNA in plasma exocrine and selected 16 strong correlation features miRNA by Lasso logistic regression. We assessed the performance of 16 miRNA for early detection and diagnosis of BC by constructing different machine learning algorithm models. The biological significance of 16 characteristic miRNAs was evaluated by bioinformatics analysis. Overall, these data highlight the value of exo-miRNA as a biomarker for BC. They may be used for early detection and diagnosis of BC in future clinical practice.

## 2. Material and Methods

### 2.1. Patients and Sample Collection

96 participants (56 patients with BC and 40 healthy subjects) who voluntarily provided informed consent were enrolled at the First People's Hospital of Foshan from January 2021 to December 2021. All patients underwent pathological biopsy to confirm the diagnosis of BC and none of the patients had received any treatments. Patients with systemic diseases or other infectious diseases were excluded. Healthy subjects were recruited among healthy adults who took routine health examinations at the First People's Hospital of Foshan and did not have any type of cancers. This study was carried out in accordance with the Declaration of Helsinki, and the study protocol was approved by The Human Investigation and Ethical Committees of the First People's Hospital of Foshan. The tubes with 10 mL ethylene diamine tetraacetic acid (EDTA) were used to gather the fasting blood samples of participants. The samples were centrifuged at low temperature and the collected supernatant was stored at -80°C.

### 2.2. Exosome Isolation and Size Determination

The isolation method of exosomes is detailed in the study of Thery and Amigorena [17]. In short, the separated exosomes were obtained by centrifugation, filtration (Merck KGaA, Darmstadt, Germany), and PBS resuspension of plasma samples, and the size and morphology of exosomes were observed by EM-2010 Transmission Electron Microscope (JEOL, Ltd., Tokyo, Japan).

### 2.3. Nanoparticle Tracking Analysis (NTA)

The equipment used in this experiment is Zetaview PMX 110 (Particle Metrix, Meerbusch, Germany). The collected exosomes were diluted with PBS to the particle concentration of 1.0 × 10^8^ ~ 1.0 × 10^9^ particles/mL, and the particle size distribution and concentration of exosomes were detected by dynamic light scattering method. The particle concentration was obtained by video analysis and normalized.

### 2.4. Western Blot

The exosome surface marker proteins CD63, CD9, and CD81 were selected for identification. The isolated exosomes were lysed to obtain protein. The denatured protein was mixed with the sample buffer and separated by polyacrylamide gel electrophoresis, then transferred to membrane, sealed, incubated with primary antibody (antibodies used in this study include CD63 mouse mAb (SC-5275, Santa Cruz, USA), CD9 rabbit mAb (#13174, CST, USA), CD81 rabbit mAb (# 56039, CST, USA), and *β*-actin rabbit mAb (#8457, CST, USA)), and incubated with the secondary antibody labeled HRP and developing using ECL agent.

### 2.5. Plasma Exosomal RNA Isolation and Small RNA Sequencing

The methods and equipment involved are equivalent to reference [18]. Plasma exosomes were isolated using ultracentrifugation. Briefly, one milliliter of plasma sample was centrifuged at 10,000× g for 30 min at 4°C to remove all cell debris. The collected supernatant was then subjected for ultrahighspeed centrifugation at 150,000× g for 70 min at 4°C. The upper liquid was discarded and then the pellet containing exosome was resuspended in 200 *μ*L PBS. Total RNA including miRNA was isolated from plasma exosome by miRNeasy Serum/Plasma Kit (QIAGEN). The quantification and size distribution of the extraction were analyzed by Qubit V.4.0 and Agilent Bioanalyzer 2100, respectively. Quantified RNA was subjected for sequencing library preparation using NEBNext Small RNA Library Prep Set for Illumina (NEB Biolabs). Purified small RNA cDNA library was quantified by Qubit V.4.0 and the size distribution was analyzed on Agilent Bioanalyzer 2100, followed by sequencing on Illumina HiSeq4000 platform.

### 2.6. Model Establishment

In this study, there were 96 samples of primitive exosomes miRNA data, including 56 BC patients (T) and 40 normal samples (N). The miRNA Count matrix of 96^∗^2504 was obtained by sequencing and data processing. We set up a training set (72 samples) and a test set (24 samples) by randomly assigning BC samples. The construction and verification of the model were completed by training set and test set, respectively. Four mainstream machine learning algorithms were used to build the model, including Lasso, SVM, GBDT, and Random Forest. The performance measurement of the model was evaluated by ROC curve, and the evaluation index was AUC. The accuracy was general when AUC between 0.5-0.7, AUC between 0.7-0.9 had a certain accuracy, and AUC greater than 0.9 had a higher accuracy.

### 2.7. Analysis of RNA Sequencing Data and miRNA Expression Levels

Following sequence alignment, known and novel microRNAs were identified using the miRDeep2 software algorithm. The miRNA expression levels were estimated by the number of reads per million and the relative miRNA expression levels were analyzed using the DEG seq method [19]. miRNAs with read counts per million mapped reads (CPM) ≥5 in at least 20% of all samples were identified as expressed miRNAs [18]. Differential expression analysis of miRNAs between the two groups were defined as having a fold change ≥2 and a false discovery rate (FDR) adjusted *P* value of <0.05. All data analysis and visualization of the differentially expressed genes were conducted using R 3.3.1 (http://www.r-project.org).

### 2.8. Prediction of miRNA Target Genes and their Molecular Pathways

Target genes of differentially regulated miRNAs were predicted using the starBase database, an integrative database for prediction of human functional microRNA targets [20]. The target gene prediction network of the 16 differentially expressed miRNAs was plotted using the Cytoscape software. The target genes were analyzed in terms of Gene Ontology (GO) [21] functional annotation and Kyoto Encyclopedia of Genes and Genomes (KEGG) [22] pathway enrichment analysis using an R package named clusterProfiler.

### 2.9. Statistical Analyses

The difference of miRNA expression level in exosomes between cases and controls was determined using Mann–Whitney U test. All statistical analyses were performed using SPSS version 23.0 (IBM Corporation, Armonk, NY, USA) and GraphPad Prism 7.0 (GraphPad Software, Inc., La Jolla, CA, USA). *P* < 0.05 was considered to indicate a statistically significant difference. The glmnet package (version number: 4.0-2) R language was used for further screening of Lasso regression; the Lambda (*λ*) value with the smallest standard error was selected to construct the optimal Lasso regression model, and the regression coefficients (*β*) factors not equal to 0 were included in the multivariate logistic regression model. SVM, random forest, and other methods all use the corresponding package of the R language. The package “pROC” (version number: 1.16.2) was used to draw the nomogram to predict the receiver operating characteristic (receiver operating characteristic, ROC) curve and calculate its area under the curve (area under ROC curve, AUC).

## 3. Results

### 3.1. Characteristics of Subjects

The clinical features of 56 BC patients and 40 healthy subjects were shown in Table 1. There was no significant difference in age distribution between BC patients and healthy subjects (*P* > 0.05). In 56 patients with BC, 46 patients (82.1%) were graded as tumor size T1-T2, and 10 patients (17.9%) as tumor size T3-T4; the number of patient with lymph node metastasis or nonlymph node metastasis was 35 (62.5%) or 21 (37.5%); 26 patients (46.4%) were graded as grading G1-G2, and 30 patients (53.6%) as grading G3; there were 18 patients (32.1%) with Estrogen Receptor (ER) negative and 38 patients (67.9%) with ER positive; there were 19 (33.9%) and 37 (66.1%) Progesterone Receptor- (PR-) negative and positive patients, respectively; there were 46 (82.1%) and 10 (17.9%) HER2-negative and positive patients, respectively.

### 3.2. Identification of Plasma Exosomes from Patients with Breast Cancer

**Figure 1:**
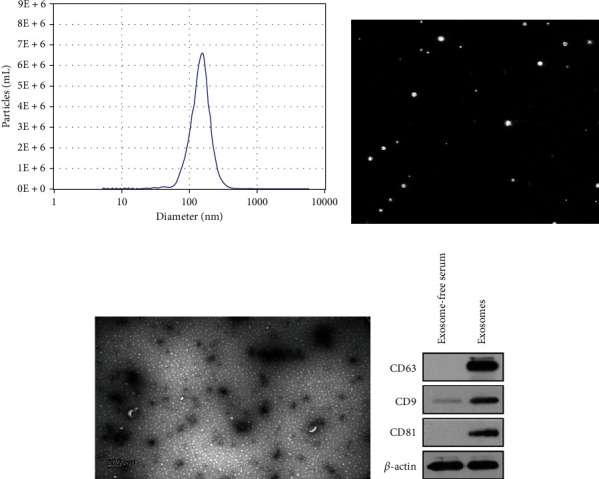
Identification of exosomes isolated from serum. (a) NanoSight analysis for serum exosomes. The average concentration and size of particles are 6.8 × 10^7^ particles/mL and 154.68 nm. (b) Observation of exosome morphology using Laser Scattering Microscopy. (c) Transmission Electron Microscopy images of isolated exosomes. Scale bar = 200 nm. (d) Using *β*-actin as an internal reference, we detected the expression levels of three exosomal protein markers, CD63, CD9, and CD81 by Western Blot.

The exosomes were successfully isolated from BC and normal controls by differential ultracentrifugation and identified with different methods. Nanoparticle tracking analysis showed that the diam of extracellular vesicles (EVs) was about 50~100 nm, and the concentration was 6.8 × 10^7^ particles/mL (Figure 1(a)), video capture of exosome movement is shown in Figure 1(b). Transmission Electron Microscopy (TEM) displayed that the isolated EVs were spheres about the same size as the exosomes (Figure 1(c)). The expression levels of three exosome marker proteins were detected by Western Blot. In the exosomes isolated from plasma, we observed the expression of CD63, CD9, and CD81 (Figure 1(d)).

### 3.3. Prediction Model Based on Circulating Exosomal miRNAs

**Figure 2:**
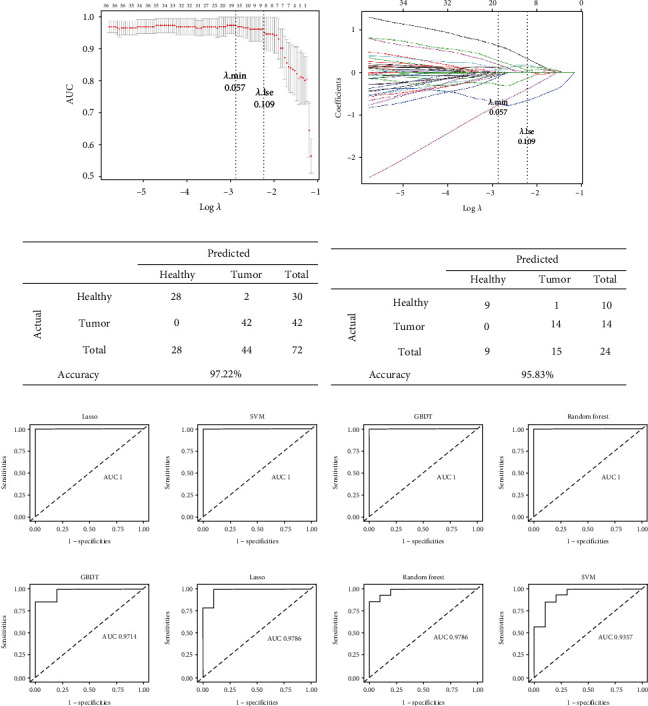
Machine learning-based diagnostic using 16 exo-miRNAs. (a) We used the R software glmnet package with the parameter family set to binomial to implement Lasso logistic regression and selected strongly correlated features. Using 5-fold cross-validation, the best performance was obtained at the highest point of the curve AUC and the penalty term coefficient Lambda. Min (0.057). Lambda.lse (0.109) is to choose a simpler model without significantly reducing the performance of the model. (b) This figure is a penalty plot of 1962 miRNA coefficients. As the penalty coefficient Lambda changes, the coefficients of more and more variables are compressed to 0, and 16 miRNAs are selected when Lambda is 0.057. (c,d) These pictures show the distribution of the training set (c) and test set (d) in different categories of samples and the accuracy of Lasso regression. (e,f) The ROC curves of the training set (e) and the test set (f) in different models, the area under the curve represents the model AUC value.

The analysis of Lasso logistic regression was performed by R software glmnet package, and the optimal *λ* value was determined through cross-validation. As shown in Figure 2(a), the penalty term coefficient Lambda = 0.057 (Lambda. Min) achieves the best performance and there were 16 miRNAs selected into variables at this time. Lambda.lse was to select a simpler model without significantly reducing the performance of the model.  Figure 2(b) was a penalty plot of 1962 miRNA coefficients. The variations of penalty coefficient Lambda made more and more variable coefficients compressed to 0.16 miRNAs were selected when Lambda was 0.057.  Figure 2(c) showed the distribution of different categories of samples in the training set, while Figure 2(d) showed the distribution of different categories of samples in the test set. In addition, the Figures 2(c) and 2(d) also showed the accuracy of Lasso regression. The results showed that the model could show high accuracy in both training set and test set. We further constructed the model using the selected 16 characteristic miRNAs. Taking the tumor sample as the concern class (positive example), Figures 2(e) and 2(f) were the ROC curves of the training set and the test set in different models, and the diagnostic efficacy of each model was shown in Table 2. The results showed that there was an excellent diagnostic performance of these four models, and the AUC values of Lasso, SVM, GBDT, and Random Forest models in the training set were 1, and the AUC values in the test set were 0.979, 0.936, 0.971, and 0.979, respectively (Table 2).

### 3.4. Expression of Exosomal miRNAs in Training Set and Test Set and Prediction of **their Target Genes**

**Figure 3:**
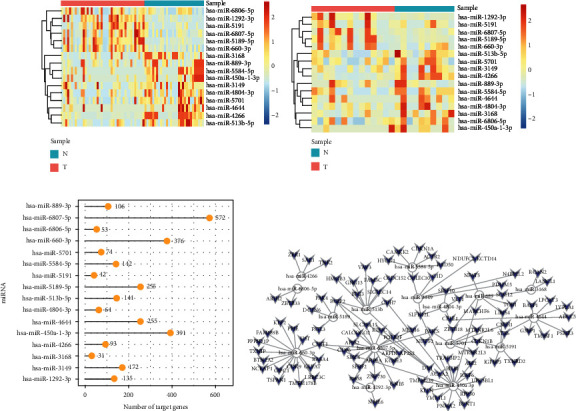
Expression of 16 miRNAs in training set and test set and prediction of their target genes. (a) Heatmap showing the expression of 16 miRNAs in the training set in tumor and normal samples. (b) Heatmap showing the expression of 16 miRNAs in the test set in tumor and normal samples. (c) The bar plot shows the total number of genes targeted by each of dysregulated miRNAs. (d) Target gene prediction network of differentially expressed miRNAs.

Further visual analysis was made on the expression of 16 miRNAs selected by Lasso in the normal group and the tumor group. The heatmaps shown in Figures 3(a) and 3(b) showed the expression of 16 miRNAs in the normal and the tumor groups of the training set and the test set, respectively. The results showed that there were 6 upregulated and 10 downregulated miRNAs in the training set, while 5 upregulated and 11 downregulated miRNAs in the test set (Supplementary Table 1). Next, we verified our results through the GEO databases, as shown in Supplementary Figure 1, miR-1292, miR-5189, and miR-660 were upregulated in the tumor group, while miR-4804-3P, miR-5701, miR-889, miR-513b, and miR-450a were downregulated in the tumor group, which was consistent with our results. Based on 16 characteristic miRNAs, each miRNA target gene was predicted using the starBase database. The number of each miRNA target gene was shown in Figures 3(c) and 3(d) which showed the target gene prediction network of the 16 differentially expressed miRNAs plotted using the Cytoscape software.

### 3.5. Biological Function Prediction of 16 miRNAs

**Figure 4:**
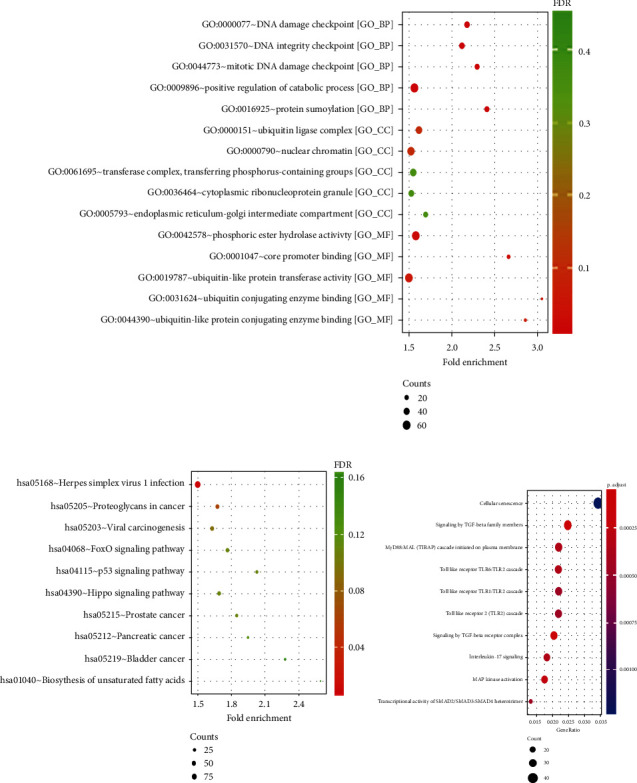
Prediction of biological functions of 16 miRNAs. (a–c) We performed GO function annotation (a), KEGG (b), and Reactome (c) pathway enrichment analysis on 16 characteristic miRNA target genes and listed the top 10 enrichment items with the most significant statistical significance.

Go function annotation was made for miRNA target genes to explore the biological significance represented by each miRNA. The results showed that 16 characteristic miRNAs were enriched in biological processes such as DNA damage checkpoint, DNA integrity checkpoint, and mitotic DNA damage checkpoint, in cell composition such as ubiquitin, ligase complex, nuclear chromatin, and transfer complex, transferring phosphorus containing groups, and in phosphoric ester hydrogen activity, core promoter binding, and ubiquitin like protein transfer activity (Figure 4(a)). KEGG pathway enrichment analysis found that herpes simplex virus 1 infection, proteoglycans in cancer and viral carcinogenesis were the enrichment entries of 16 characteristic miRNAs (Figure 4(b)). Reactome pathway enrichment results showed that 16 characteristic miRNAs were mainly enriched in signaling by TGF-*β* receptor complex, signaling by TGF-*β* family members and Toll-like receptor family (Figure 4(c)). Furthermore, we performed the GO and KEGG enrichment analyses of target genes of 6 upregulated miRNAs and 10 downregulated miRNAs, respectively (Supplementary Figures 2–3).

## 4. Discussion

MicroRNA (miRNA) in plasma exosomes has been reported to be a potential biomarker in many cancers. However, its diagnostic value in BC needs to be further determined. In our study, 96 participants (56 patients with BC and 40 healthy subjects) were randomly divided into 72 samples in the training set and 24 samples in the test set. The expression of exosome miRNAs in plasma of all subjects was evaluated by RNA sequencing technology. Finally, 16 characteristic miRNAs were selected by using Lasso logical regression, and different models were constructed to evaluate the diagnostic performance of 16 characteristic miRNAs in training set and test set. Furthermore, we used bioinformatics analysis to predict 16 crucial miRNA signaling pathways involved in BC, which linked our biomarkers with the underlying mechanisms of BC.

In recent years, many related studies have applied machine learning models to the diagnosis and prognosis evaluation of BC. As early as 2005, there were researchers using artificial neural networks and decision trees algorithms and logistic regression to develop large datasets to establish a prognostic model for BC patients, and this is the first time that machine learning algorithm has been applied to the study of BC patients' prognosis evaluation [23]. Other studies have shown that the prognosis of invasive BC could be predicted well by machine learning models [24]. Exosomes contain many types of proteins, DNA, RNA, and other substances, which are promising biomarkers of cancer for early diagnosis. In addition, in vitro and in vivo miRNAs in plasma have been shown to be differentially expressed. In this study, we used plasma exosomes miRNAs as biomarkers to construct a model for detection of BC. Among the four models we constructed, it is preliminarily considered that Lasso and Random Forest have the best properties, and their AUC values are 0.979, higher than SVM and GBDT. The models based on 16 miRNAs in this study have high diagnostic value in the early diagnosis of BC, and the sensitivity of the training set and test set of Lasso, GBDT, and Random forest models was as high as 90%, which was superior than routine tumor biomarkers CA153, CA125, and CEA (their sensitivities were 14.6%, 14.6%, and 81.3%, respectively) [25]. It is also reported that the combined detection of CA153 and other exosomal miRNA can improve the sensitivity of BC diagnosis [26]. Therefore, the diagnostic value of the combination of the 16 miRNAs and CA153 is worth to study in the future, and we will also enhance the performance of the predictive model through increasing the sample size.

It has been reported that miR-889-3p, miR-660-5p, miR-513b-5p, and miR-450a are related to the proliferation, invasion, and migration of BC, and high expression of miR-660-5p was closely related to lymph node metastasis, advanced TNM stage, and vascular invasion of BC tumors, which might be a promising target for BC treatment [27–30]. Chen et al. have reported the role of miR-4644 in prediction of therapeutic responses and suggest that it could serve as valuable sources for biomarker detections and optimal chemotherapeutic choices for BC patients [31]. However, the role of the remaining 11 miRNAs in BC has not been reported yet. This study is the first to report the relationship between these 11 miRNAs and BC. In order to determine the biological function of 16 characteristic miRNA selected in BC from this study, we used starBase database to predict target genes and carried out bioinformatics analysis. The results showed that herpes simplex virus 1 infection and proteoglycans in cancer pathway were the main enrichment entries of the target genes of 16 characteristic miRNAs. It has been reported that HSV-1 infection is a key factor in the progression of BC; [32, 33] more interestingly, HSV-1 is an oncolytic virus that can kill tumor cells, and it has been found that the attenuated HSV-1 clone, namely HF10, can lysate human and mouse BC cells in vitro and is expected to be further used in the treatment of BC [34]. The above data fully supported the close relationship between HSV-1 and BC, and our data showed that 16 characteristic miRNA target genes were significantly enriched in HSV-1 infection, proving that it had important biological significance. Another study reported that TGF-*β* signal pathway regulates the EMT process, tumor microenvironment, and the stemness in BC cells; [35] TLR2 and TLR4 were highly expressed in serum of BC patients and were associated with multiple clinicopathological parameters, it might be a potential diagnostic biomarker for BC [36]. Reactome pathway enrichment analysis in this study showed that 16 characteristic miRNA target genes were enriched in signal pathways mediated by TGF-*β* and Toll-like receptors, which was consistent with the results of reported studies, and it further illustrated the importance of 16 characteristic miRNAs in the development of BC. One of the limitations of this study was related to the sampling quantities, especially for the construction of prediction models. Consequently, the actual accuracy of the prediction might not be as vigorous as it seems. In our next step, the samples will be expanded and the predictive model will be further refined accordingly.

Although the biological and functional characteristics of exosomes in BC are becoming more clear, there are still some difficulties in applying exosome detection to clinical diagnosis. The exosomes obtained from the body originate from different cell types. The traditional analysis methods are not enough to identify the specific source of exosomes. It is imperative to explore an efficient, rapid, and economical method to determine the source of exosomes in the future.

## 5. Conclusions

To sum up, our research has identified a set of exo-miRNAs that can be used to detect BC. This will provide a new powerful basis for early diagnosis of BC.

## Figures and Tables

**Table 1:** Clinical characteristics of patients with breast cancer included in the study.

Characteristic	Breast cancer no. (%)	Control no. (%)
Total	56	40
Age		
<50	27 (48.2)	18 (46.6)
≥50	29 (51.8)	22 (53.1)
Tumor size		
T1-2	46 (82.1)	—
T3-4	10 (17.9)	—
Lymph node metastasis		
(-)	35 (62.5)	—
(+)	21 (37.5)	—
Grading		
G1-2	26 (46.4)	—
G3	30 (53.6)	—
ER		
(-)	18 (32.1)	—
(+)	38 (67.9)	—
PR		
(-)	19 (33.9)	—
(+)	37 (66.1)	—
HER2		
(-)	46 (82.1)	—
(+)	10 (17.9)	—

**Table 2:** Model diagnostic performance based on Lasso algorithm.

Diagnostic value	Training set				Test set			
	Lasso	SVM	GBDT	Random Forest	Lasso	SVM	GBDT	Random Forest
AUC	1.000	1.000	1.000	1.000	0.979	0.936	0.971	0.979
Accuracy	0.972	0.986	1.000	1.000	0.958	0.833	0.875	0.917
Sensitivity (%)	0.933	0.967	1.000	1.000	0.900	0.700	0.900	0.900
Specificity (%)	1.000	1.000	1.000	1.000	1.000	0.929	0.857	0.929

## Data Availability

The datasets generated and/or analyzed during the current study are available in the GEO database (https://www.ncbi.nlm.nih.gov/geo/).

## Supplementary Materials

Supplementary 1 Supplementary Table 1. Differentially expressed miRNAs in the normal and tumor groups of the training set and the test set.

Supplementary 2 Supplementary Figure 1. The expression of differential miRNAs in breast cancer and normal groups from GEO database. Supplementary Figure 2. Prediction of biological functions of target genes of 6 upregulated miRNAs. Supplementary Figure 3. Prediction of biological functions of target genes of 10 downregulated miRNAs.
