# Supplementary material for: A Breast Cancer Prediction Model Based on a Panel from Circulating Exosomal miRNAs
Source: Biomed Res Int. 2022 Oct 20;2022:5170261. doi: 10.1155/2022/5170261 (PMC9615554; doi:10.1155/2022/5170261)
Supplement: Supplementary 1 — Supplementary Table 1. Differentially expressed miRNAs in the normal and tumor groups of the training set and the test set. [file 5170261.f1.docx]

**Supplementary** **Tables**

**Supplementary table 1 Differentially expressed miRNAs in the normal and tumor groups of the training set and the test set**

| miRNA | Training set  Log FC FDR P value | Test set  Log FC FDR P value |
| --- | --- | --- |
| hsa-miR-6806-5p | 0.521 0.054 0.024 | -0.681 0.326 0.124 |
| hsa-miR-1292-3p | 1.109 <0.0001 <0.0001 | 0.916 0.160 0.029 |
| hsa-miR-5191 | 0.933 0.001 <0.0001 | 0.199 0.786 0.630 |
| hsa-miR-6807-5p | 0.790 0.003 <0.0001 | 0.940 0.173 0.036 |
| hsa-miR-5189-5p | 1.060 <0.0001 <0.0001 | 0.928 0.164 0.031 |
| hsa-miR-660-3p | 0.956 0.001 <0.0001 | 0.412 0.574 0.358 |
| hsa-miR-3168 | -0.956 0.001 <0.0001 | -0.731 0.240 0.066 |
| hsa-miR-889-3p | -0.661 0.014 0.004 | -0.123 0.872 0.764 |
| hsa-miR-5584-5p | -1.058 <0.0001 <0.0001 | -0.743 0.259 0.077 |
| hsa-miR-450a-1-3p | -0.801 0.003 0.001 | -0.565 0.383 0.165 |
| hsa-miR-3149 | -0.493 0.069 0.033 | -0.901 0.186 0.042 |
| hsa-miR-4804-3p | -0.717 0.008 0.002 | -0.753 0.265 0.082 |
| hsa-miR-5701 | -0.699 0.009 0.002 | -0.704 0.316 0.116 |
| hsa-miR-4644 | -0.718 0.008 0.002 | -0.766 0.274 0.088 |
| hsa-miR-4266 | -1.295 <0.0001 <0.0001 | -1.376 0.034 0.001 |
| hsa-miR-513b-5p | -0.738 0.006 0.001 | -0.703 0.261 0.078 |
