# Supplementary material for: A Breast Cancer Prediction Model Based on a Panel from Circulating Exosomal miRNAs
Source: Biomed Res Int. 2022 Oct 20;2022:5170261. doi: 10.1155/2022/5170261 (PMC9615554; doi:10.1155/2022/5170261)
Supplement: Supplementary 2 — Supplementary Figure 1. The expression of differential miRNAs in breast cancer and normal groups from GEO database. Supplementary Figure 2. Prediction of biological functions of target genes of 6 upregulated miRNAs. Supplementary Figure 3. Prediction of biological functions of target genes of 10 downregulated miRNAs. [file 5170261.f2.docx]

**Supplementary** **Figures:**


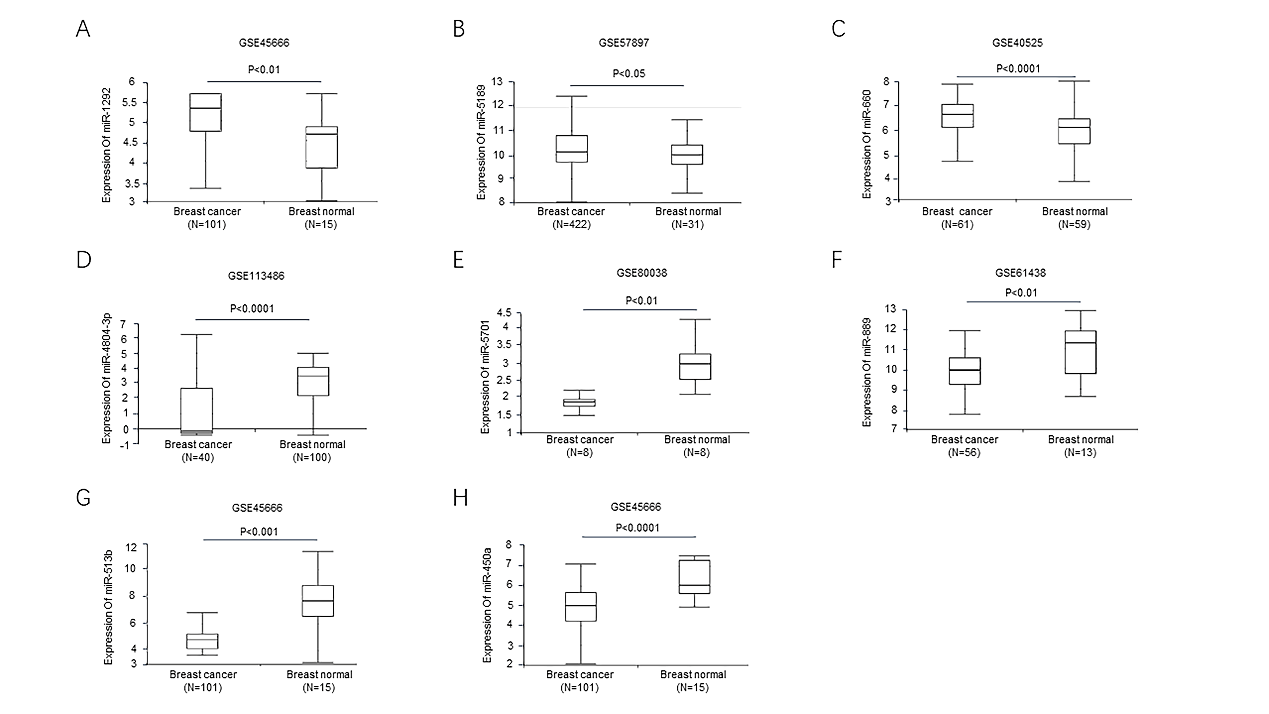


**Supplementary Figure 1:** The expression of differential miRNAs in breast cancer and normal groups from GEO database. (**A-C**) miR-1292, miR-5189 and miR-660 were dramatically upregulated in breast cancer group in the GEO database. (D-H) miR-4804-3P, miR-5701, miR-889, miR-513b and miR-450a were dramatically downregulated in breast cancer group in the GEO database.


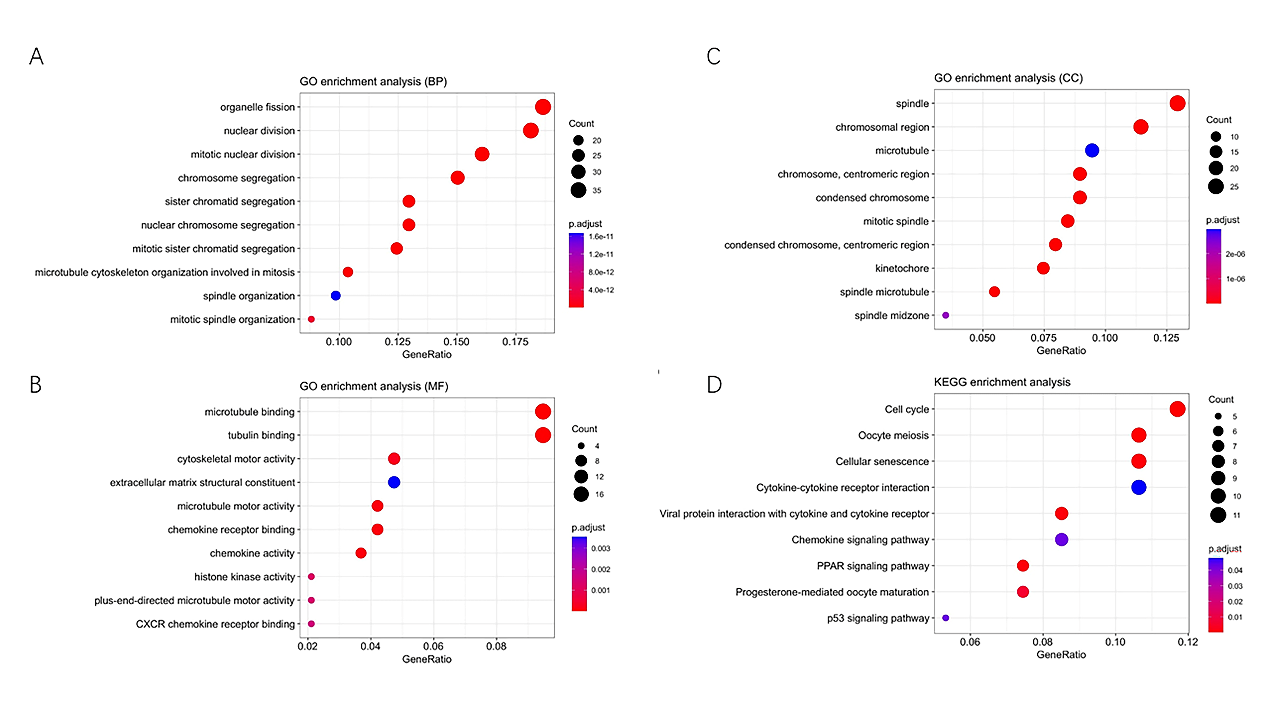


**Supplementary Figure 2:** Prediction of biological functions of target genes of 6 up-regulated miRNAs. (A-C) The GO enrichment analysis of target genes of 6 up-regulated miRNAs. (D) The KEGG enrichment analysis of target genes of 6 up-regulated miRNAs.


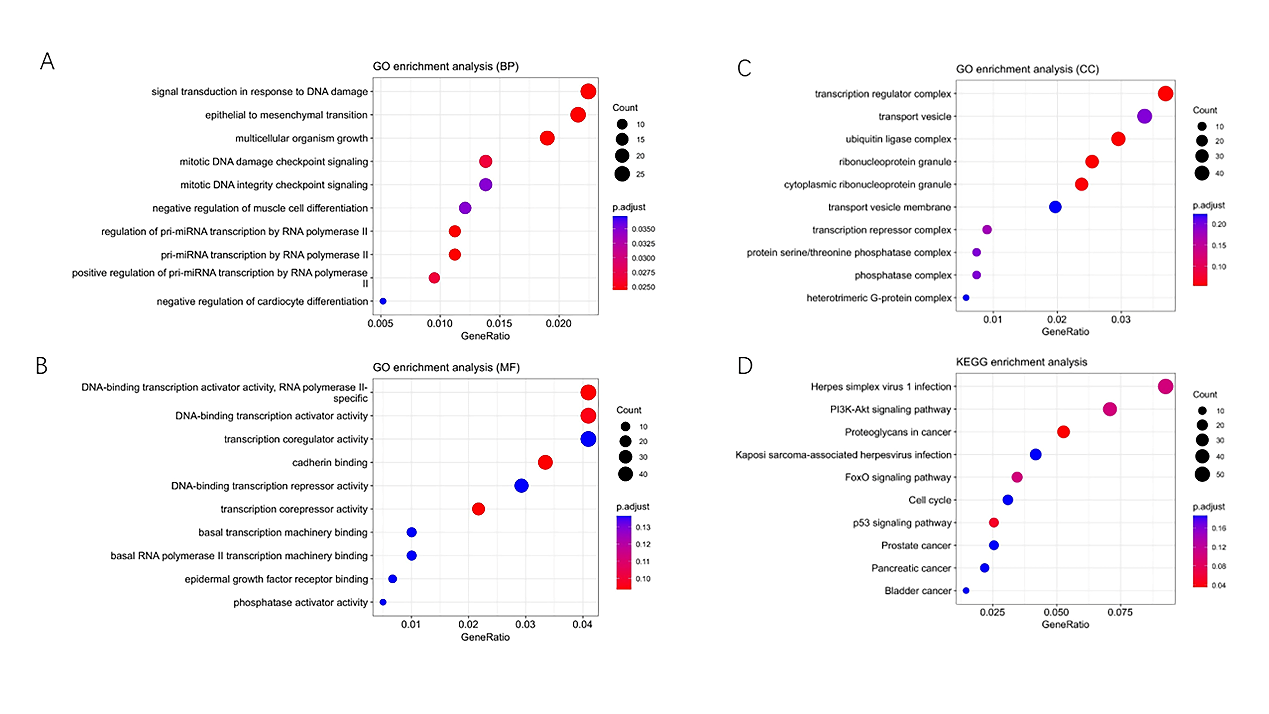


**Supplementary Figure 3:** Prediction of biological functions of target genes of 10 down-regulated miRNAs. (A-C) The GO enrichment analysis of target genes of 10 down-regulated miRNAs. (D) The KEGG enrichment analysis of target genes of 10 down-regulated miRNAs.
